# An evolutionary game for the behavior of third-party evaluators in pension public–private partnership incorporating public participation

**DOI:** 10.1038/s41598-023-47369-1

**Published:** 2023-11-23

**Authors:** Xianghua Yue, Shahzad Khan Durrani, Shikuan Zhao, Fuda Li

**Affiliations:** 1 School of Economics and Management, Xiangnan University, Chenzhou, 423000 People’s Republic of China; 2 School of Management and Economics, Beijing Institute of Technology, Beijing, 100081 People’s Republic of China; 3 School of Public Policy and Administration, Chongqing University, Chongqing, 400044 People’s Republic of China; 4 Business School, Hunan Normal University, Changsha, 410000 People’s Republic of China

**Keywords:** Evolution, Health care, Health occupations, Engineering, Mathematics and computing, Optics and photonics

## Abstract

This study analyzes the impact of public participation on the choice of third-party evaluators' behavior strategies during the service quality supervision process of China's pension public–private partnership project. An evolutionary game model between third-party evaluators and government regulators is developed, wherein the evolution rule of the two sides and public participation’s influence on their behavior under the two different conditions are analyzed, and a numerical simulation is carried out using MATLAB 2016a. It is found that third-party evaluators may choose the *false evaluation* strategy without public participation because of the inducement of rent-seeking or insufficient government punishment when the regulatory revenue of the government regulatory agencies is less than the regulatory cost. In contrast, in the case of public participation, the *true evaluation* strategy is chosen with an improvement in the level of public participation or an increase in reputation incentive. This suggests the construction and improvement of a third-party evaluation system, which shows that the construction of the service quality supervision system in China’s pension PPP project has a large operating space.

## Introduction

Public–Private Partnership (PPP) represents a pioneering collaborative model designed to integrate private investors into the realm of public services and infrastructure development, a practice increasingly adopted by nations^1–3^. In China, the government has enthusiastically embraced the PPP model within the pension industry, attaining notable milestones spanning the entire life-cycle of pension PPP projects, from inception to delivery and ongoing operation since 2014. However, limitations regarding regulatory capacity, skilled personnel, regulatory funding, and other resources have prompted Chinese government bodies to enlist third-party assessment agencies in supervising the service quality of pension PPP projects. These agencies play a pivotal role in linking operational subsidies of pension PPP projects to their assessment outcomes. This symbiotic relationship serves to mitigate the constraints stemming from regulatory resource scarcity, while simultaneously generating novel regulatory pressures and deterrent effects.

The term "third-party evaluators" alludes to academic institutions or professional evaluation entities possessing extensive experience in pension service research and adeptness in evaluating the quality of pension services. Nonetheless, within the PPP framework, characterized by information asymmetry^4^, third-party evaluators may encounter biases induced by economic incentives from private investors during the evaluation process, potentially resulting in rent-seeking behaviors and ultimately leading to unscrupulous evaluations. The challenge further manifests as government regulators grapple with an incomplete understanding of third-party evaluation behavior, thereby engendering potential government failures.

As China's pension PPP projects undergo rapid expansion, the imperative of good governance becomes inexorable for their long-term viability. Embedded within the rubric of good governance lies the essential element of public participation^5^. To forestall the dissemination of erroneous information by third-party assessment institutions, governmental oversight, and management of these entities during project service quality evaluations becomes paramount. Chinese government regulators must galvanize public engagement, encompassing ordinary citizens, the news media, and various societal groups, to partake in evaluations and oversight. The overarching objective of pension PPP projects resides in furnishing the public with high-quality, cost-effective pension services. Given that public resistance frequently emerges as a critical factor contributing to project failures^6,7^, it assumes a pivotal role in ensuring smooth project execution^8–11^. The sustainability of pension PPP projects hinges upon public endorsement of service quality. Consequently, the public can contribute directly or indirectly (via complaints and reports), thus becoming valuable sources of governmental supervision.

The critical inquiry revolves around whether public participation enhances the reliability of third-party evaluations in the oversight of China's pension PPP projects. Moreover, how can a judicious oversight strategy be crafted that incorporates public participation and incentivize third-party evaluators towards greater responsibility?

From a qualitative perspective, it is imperative to establish effective regulatory mechanisms and precise incentive structures to safeguard the veracity and objectivity of third-party evaluation reports. Initially, evaluators must undergo oversight through legal frameworks, standard establishment, public engagement, and other measures to obviate the misuse of their regulatory and evaluative informational advantages in pursuit of rent-seeking and improper interests. Additionally, the establishment of a reputation-based mechanism is imperative for incentivizing third-party evaluators^12^. This mechanism would steer them towards objectivity, robust evaluation of information quality, and the disciplining of those who fail in their duties.

However, the challenge lies in the validation of these propositions from a mathematical vantage point. Within the ambit of pension PPP project supervision, government regulators and third-party evaluators grapple with information asymmetry, culminating in a dynamic, iterative game. This dynamic stems from information incompleteness and is compounded by the cognitive and computational limitations of both third-party evaluators and government regulators. Given the bounded rationality inherent in these decision-makers, we endeavor to harness evolutionary game theory (EGT) as an analytical tool. EGT will be employed to dissect how three pivotal factors impact the decision-making proclivities of third-party evaluators: (1) the degree of public participation, (2) the incentive structure revolving around reputation, and (3) the application of punitive measures. This study endeavors to unravel the mutual evolutionary dynamics of third-party evaluators vis-à-vis these factors and to proffer a well-grounded supervision strategy that incorporates public engagement, compelling third-party evaluators toward greater accountability.

## Rationale for applying evolutionary game theory (EGT)

### Academic principles

EGT stands as an exceptionally apt framework for modeling and dissecting complex interactions among strategic agents^13^. Within PPP projects, these interactions transcend mere evaluation entities, encompassing government regulators, private investors, and the public. Notably, these stakeholders often harbor divergent objectives, engendering recurrent and intricate interactions. EGT, with its dynamic and evolving modeling capabilities, provides an indispensable tool for unraveling the nuances of these multifaceted dynamics.

EGT's unique prowess lies in its capacity to capture the adaptive nature of behavior and the learning process over time^14^. In the specific context of third-party evaluators, their strategies exhibit a propensity for evolution, influenced by past experiences and the consequences of prior evaluations. This adaptability assumes paramount significance when deciphering how these evaluators respond to shifts in regulatory policies or variations in the degree of public involvement.

EGT offers an established framework for analyzing strategic decision-making processes^15^. Third-party evaluators confront strategic dilemmas, oscillating between the delivery of precise assessments that benefit public welfare and the temptation of engaging in rent-seeking behavior to maximize their profits. Concurrently, government regulators face strategic crossroads regarding supervision strategies. EGT empowers us to model these strategic choices and illuminate their far-reaching consequences.

EGT's utility extends to capturing the intricate dynamics of agent behavior in response to incentives and penalties^16,17^. A compelling illustration emerges from a study involving the modeling of interactions among traffic management departments (TMD), drivers, and pedestrians at crosswalks. This study underscores that, under penalty-incentive control or with robust TMD supervision, drivers willingly yield to pedestrians, potentially enhancing pedestrian safety—a paradigm particularly pertinent to PPP projects characterized by public scrutiny, concerns about reputation, and the allure of rewards or sanctions.

PPP projects, particularly within the pension sector, carry profound policy implications^18^. Government policies, regulatory mechanisms, and stakeholder conduct wield substantial influence over project outcomes and sustainability. EGT offers an indispensable lens through which policymakers and researchers can fathom the consequences of diverse policy choices.

Empirical evidence substantiates the applicability of EGT across a gamut of real-world scenarios encompassing social and economic systems^19^. Noteworthy research endeavors have harnessed EGT to investigate cooperation, competition, and the emergence of intricate behaviors in diverse contexts.

For instance, resource allocation mechanisms fostering cooperation within well-mixed populations have been examined, effectively addressing second-order free-rider problems^19^. This research underlines the efficacy of allocating resources based on individual contributions and rewarding cooperative behavior, underscoring the relevance of such cooperative strategies in intricate socio-economic environments.

Similarly, investigations have delved into the role of monitoring, reporting, and sanctioning mechanisms in enhancing cooperation amidst collective risk dilemmas^18^. These studies have illuminated the significance of these mechanisms in comprehending cooperation dynamics, further underscoring the applicability of EGT.

Additionally, explorations into resource allocation strategies in collective-risk social dilemmas have divulged critical insights, demonstrating the emergence of win–win scenarios promoting cooperation and the sustenance of shared resources under specific conditions^20^^.^

A remarkable study has probed the efficacy of employing positive and negative incentives in governing common resources under risky conditions, identifying a local sanctioning scheme combined with pure rewards as the most potent strategy. This approach stimulates populations towards higher levels of cooperation across diverse parameters and institutional contexts, providing invaluable insights for sustainable resource management policies^21^.

In summary, the adoption of EGT in understanding the conduct of third-party evaluators within pension PPP projects, particularly in the context of public participation, is richly justified. EGT's capacity to model intricate interactions, elucidate adaptive behaviors, dissect strategic decision-making, and provide a rigorous framework for comprehending real-world phenomena finds ample resonance within existing academic research. This alignment underscores the aptness and relevance of EGT as an invaluable tool for advancing our understanding of the intricate dynamics governing PPP projects.

### Literature support

EGT is dedicated to research based on the idea that players cannot fully grasp the entirety of the information, and their decision-making will change based on updated knowledge^22,23^. This kind of game theory has achieved success in research on different social fields, such as vaccine dilemma^24^, sustainable energy development^25^, environmental pollution^26^, sustainable tourism^27^, rights^28^, and Social physics^29^. There are also some scholars using the EGT to research PPP projects under supervision from different perspectives. Some emphasized the importance of punishment in the supervision of PPP projects^30–33^, and some concluded that punishment would be ineffective for private investors who had violated regulations if government regulators failed to perform their duties^34^. Others have discussed the importance of public participation and reputation in project supervision^35^. However, these studies take private investors as the regulated objects, ignoring other stakeholders’ important roles, such as third-party evaluators and the public, in the PPP project supervision game mechanism. The behavioral interactions between third-party evaluators and government regulators have a meaningful impact on project supervision. Analyzing the evolution process and influencing third-party evaluators’ behavior from dynamic and quantitative perspectives is of practical significance.

The existing literature exhibits discernible gaps that, while acknowledging the intricate nature of public participation within our evolutionary game model, underscore the significance of our research. Our contributions are twofold. Firstly, we scrutinize the dynamic evolutionary trajectory and the influencing variables governing third-party evaluators' conduct without public engagement, delving into micro-level intricacies. This endeavor bestows upon us a fresh lens through which we can optimize governmental oversight mechanisms. Secondly, we amalgamate evolutionary game theory (EGT) with numerical simulation methodologies, unraveling the governing dynamics of mutual evolution between government regulators and third-party evaluators. We also evaluate how public participation steers the behavioral strategies of both parties, thus elevating the current state of research. In summation, our research strives to proffer a novel perspective and theoretical road-map. Our objective is to optimize governmental oversight mechanisms, foster confidence in third-party evaluations, and achieve the overarching goal of sustainable development for pension Public–Private Partnership (PPP) projects by instituting a third-party evaluation mechanism integrated with public participation.

### Structural framework

The subsequent sections of this paper are structured as follows:

“Rationale for applying evolutionary game theory (EGT)” section: Offers an incisive analysis of the evolutionary game that unfolds between government regulators and third-party evaluators when public participation is absent. This section delves into the intricate dynamics of their interactions and provides a novel perspective on enhancing governmental oversight mechanisms.

"Construction and analysis of models without public participation" section: Investigates the evolutionary game between government regulators and third-party evaluators, taking into account the pivotal element of public participation. It delves into the behavioral strategies adopted by these stakeholders in response to public engagement, shedding light on their dynamics.

"Construction and analysis of the public participation model" section: Employs numerical simulation analysis as a validating tool to assess the efficacy of our model results. This section rigorously examines the outcomes of our research, lending empirical weight to our theoretical framework.

"Numerical analysis" section: Offers conclusive insights drawn from the empirical results, thereby elucidating the policy implications that can be gleaned from our research. We conclude this section by providing recommendations for future research avenues.

This structured framework ensures a comprehensive and academically rigorous exploration of our research agenda, facilitating an enriched understanding of the complex dynamics inherent in the oversight of pension PPP projects.

## Construction and analysis of models without public participation

### Model construction

Suppose Chinese government regulators entrust third-party evaluators to regularly evaluate the service quality provided by pension PPP projects to ensure maximum public welfare. As the game model participants, it is assumed that the differences between third-party evaluators and government regulators themselves are not considered. Owing to information asymmetry, both players are bounded rationally and their objectives are different. Government regulators appeal to society’s overall interests, but third-party evaluators pursue profit maximization. The setting parameters and descriptions are as follows.

Hypothesis 1: Third-party evaluators have two strategic choices: *true evaluation* (*TE*) *and false evaluation* (*FE*)*. TE* indicates that third-party evaluators hire professional evaluators, use advanced evaluation techniques and uniform evaluation criteria, refuse to rent to private investors, issue an accurate evaluation report, etc., where $C_{{\text{t}}}$ is the cost. *FE* indicates that third-party evaluators hire amateurs for evaluation work, do not use uniform evaluation criteria and advanced evaluation techniques, accept rent-seeking from private investors, issue false evaluation reports, etc., where $C_{{\text{f}}}$ is the cost. At this time, $C_{{\text{t}}} > C_{{\text{f}}}$. $R_{t}$ is the revenue that third-party evaluators are entrusted by government regulators to assess. $R_{r}$ is the rent-seeking income from private investors to third-party evaluators of *FE*. $\alpha$(${0} \le \alpha \le {1}$) is the probability that third-party evaluators choose to rent with private investors. $F_{{\text{t}}}$ is the fine imposed by the government regulators on third-party evaluators for *FE.*

Hypothesis 2: Government regulators also have two choices: *active supervision* (*AS*) and *negative supervision* (*NS*). *AS* indicates that government regulators actively supervise and inspect third-party evaluators’ assessments, regularly comparing the data with the evaluation reports from third-party evaluators to avoid third-party evaluator violations. *NS* indicates that government regulators do not supervise and examine third-party evaluations and do not promptly compare the data with the evaluation reports. $R_{{\text{g}}}$ is the benefit to be obtained from government regulators choosing *AS* strategy, such as superior incentives for subordinates, departmental subsidies, public recognition of government regulators’ supervision, and so on.$C_{g}$ is the cost of the *AS* strategy by the government regulators.

Hypothesis 3: Under the *NS* strategy of government regulators, $\beta$(${0} \le \beta \le {1}$) is the probability of being discovered by the higher government department, and the loss of third-party evaluators for *FE*(e.g., government fines, reduced evaluation business volume, revocation of evaluation qualifications, etc.), and $\beta F_{{\text{g}}}$ is the loss of government regulators for *NS*.

Hypothesis 4: $x$(${0} \le x \le {1}$) is the probability that third-party evaluators choose the *TE* strategy; then, $1 - x$ is the probability of the *FE* strategy chosen. $y$ is the probability that government regulators choose the *AS* strategy, and $1 - y$ is the probability that the *NS* strategy is chosen.

To simplify the game model, it is assumed that government regulators can detect the *FE* of third-party evaluators under *the AS* strategy, while they cannot under *the NS* strategy.According to the above assumptions, we can obtain the evolutionary game payment matrix between third-party evaluators and government regulators without public participation, as shown in Table 1.

**Table 1:** Evolutionary game payment matrix without public participation.

Third-party evaluators	Government regulators	
	*AS* ($y$)	*NS* ($1 - y$)
*TE* ($x$)	$R_{t} - C_{t}$,$R_{g} - C_{g}$	$R_{t} - C_{t}$,$0$
*FE* ($1 - x$)	$R_{t} { + }\alpha R_{r} - C_{f} - F_{t}$,$R_{g} - C_{g}$	$R_{t} { + }\alpha R_{r} - C_{f} - \beta F_{{\text{t}}}$,$- \beta F_{g}$

### Evolutionary strategy stability analysis

From the above game matrix (Table 1), the expected revenue of *the TE* strategy selected by third-party evaluators is given by

1 $$E_{x} = y(R_{t} - C_{{\text{t}}} \, ) + (1 - y)(R_{t} - C_{{\text{t}}} \, )$$

The expected revenue of third-party evaluators choosing the *FE* strategy is given by

2 $$E_{1 - x} = y(R_{t} { + }\alpha R_{r} - C_{f} - F_{t} ) + (1 - y)\left( {R_{t} { + }\alpha R_{r} - C_{f} - \beta F_{{\text{t}}} } \right)$$

Then the average expected revenue of third-party evaluators is given by

3 $$\overline{E} = xE_{x} + (1 - x)E_{1 - x}$$

According to the Malthusian dynamic equation^36^, the replicator dynamic equation for third-party evaluators is given by

4 $$\frac{dx}{{dt}} = x(1 - x)\left( {E_{x} - E_{{1{ - }x}} } \right) = x(1 - x)[y(1 - \beta )F_{t} - (C_{t} - C_{f} - \beta F_{t} + \alpha R_{{\text{r}}} )]$$

Similarly, the replicator dynamic equation for government regulators is

5 $$\frac{dy}{{dt}} = y(1 - y)\left( {R_{g} - C_{g} + \beta F_{g} - x\beta F_{g} } \right)$$

Therefore, under the pension PPP model, the evolution of the behavior strategies of third-party evaluators and government regulators is described by a two-dimensional dynamic system *L*_*1*_ consisting of replicated dynamic Eqs. (4) and (5), that is given by

6 $$\left\{ \begin{aligned} \frac{{{\text{d}}x}}{dt} & = x(1 - x)[y(1 - \beta )F_{t} - (C_{t} - C_{f} - \beta F_{t} + \alpha R_{r} )] \hfill \\ \frac{dy}{{dt}} &= y(1 - y)(R_{g} - C_{g} + \beta F_{g} - x\beta F_{g} ) \hfill \\ \end{aligned} \right.$$

Let $\frac{{{\text{dx}}}}{dt} = 0$; and $\frac{{{\text{dy}}}}{dt} = 0$, then $(0,0)$,$(0,1)$,$(1,0)$,$(1,1)$ and $\left( {\frac{{R_{g} - C_{g} + \beta F_{g} }}{{\beta F_{g} }},\frac{{C_{t} - C_{f} + \alpha R_{r} - \beta F_{t} }}{{F_{{\text{t}}} - \beta F_{{\text{t}}} }}} \right)$ can be obtained by analyzing the equilibrium point of system *L*_*1*_. However, not all equilibrium points of system *L*_*1*_ can be an evolutionary stable strategy (ESS)^37,38^. To explore the ESS of system *L*_*1*_, we analyzed the local stability of the Jacobian matrix of two-dimensional dynamical systems according to the method proposed by Friedman^39^ and then determined the stability of each equilibrium point. The Jacobian matrix of the dynamical system *L*_*1*_ is:

7 $$J_{1} = \left[ {\begin{array}{ll} {(1 - 2x)[y(1 - \beta )F_{t} - (C_{t} - C_{f} + \alpha R_{r} - \beta F_{t} )]} & \quad {x(1 - x)(1 - \beta )F_{t} } \\ { - y(1 - y)\beta F_{g} } & \quad {(1 - 2y)(R_{g} - C_{g} + \beta F_{g} - x\beta F_{g} )} \\ \end{array} } \right]$$

If the following two conditions are satisfied simultaneously, the equilibrium point of the replicated dynamic equation is the evolutionary stability strategy (ESS).

1. $${\text{tr}}J = (1 - 2x)[y(1 - \beta )F_{t} - (C_{t} - C_{f} + \alpha R_{r} - \beta F_{t} )] + (1 - 2y)(R_{g} - C_{g} + \beta F_{g} - x\beta F_{g} ) < 0$$ (Trace condition);
2. $\det J = (1 - 2x)[y(1 - \beta )F_{t} - (C_{t} - C_{f} + \alpha R_{r} - \beta F_{t} )](1 - 2y)(R_{g} - C_{g} + \beta F_{g} - x\beta F_{g} ) + xy(1 - x)(1 - y)(1 - \beta )\beta F_{t} F_{g} > 0$ (Jacobian determinant condition)

Because there is $trJ = 0$ a local equilibrium point $\left( {\frac{{R_{g} - C_{g} + \beta F_{g} }}{{\beta F_{g} }},\frac{{C_{t} - C_{f} + \alpha R_{r} - \beta F_{t} }}{{F_{{\text{t}}} - \beta F_{{\text{t}}} }}} \right)$, it is not the equilibrium point of the system evolution stability strategy. Therefore, only ${\text{tr}}J$ and $\det J$ of the Jacobian matrix at the remaining four local equilibrium points must be considered. Next, the ESS of system *L*_*1*_ is analyzed in four cases as follows.

Case 1.: When $F_{t} < C_{t} - C_{f} + \alpha R_{r}$ and $R_{g} < C_{g} - \beta F_{g}$ at the same time, $(0,0)$ is the only stable point of system *L*_*1*_. The ESS of system *L*_*1*_ is that third-party evaluators tend to choose the *FE* strategy, and government regulators tend to choose the *NS* strategy. According to the two-dimensional dynamic system *L*_*1*_, the sum values of the equilibrium points of Jacobian matrix *J*_*1*_ are obtained. Furthermore, the local stability of system *L*_*1*_ was determined, as shown in Table 2.

**Table 2:** Local stability analysis of Case 1 and Case 2.

Balance point	Case 1			Case 2		
	${\text{tr}}J$	$\det J$	Local stability	${\text{tr}}J$	$\det J$	Local stability
$(0,0)$	$-$	$+$	ESS	$\pm$	$-$	Saddle point
$(0,1)$	$\pm$	$-$	Saddle point	$-$	$+$	ESS
$(1,0)$	$\pm$	$-$	Saddle point	$+$	$+$	Instability point
$(1,1)$	$+$	$+$	Instability point	$\pm$	$-$	Saddle point
Case 2.: When $F_{t} < C_{t} - C_{f} + \alpha R_{r}$ and $R_{g} > C_{g}$ at the same time, $(0,1)$ is the only stable point of system *L*_*1*_. The ESS of system *L*_*1*_ is that third-party evaluators tend to choose the *FE* strategy, and government regulators tend to choose the *AS* strategy. According to the two-dimensional dynamic system *L*_*1*_, the sum values of the equilibrium points of Jacobian matrix *J*_*1*_ are obtained. Moreover, the local stability of system *L*_*1*_ was determined, as shown in Table 2.
Case 3.: When $F_{t} > \frac{{C_{t} - C_{f} + \alpha R_{r} }}{\beta }$ and $R_{g} < C_{g} - \beta F_{g}$ at the same time, $(1,0)$ is the only stable point of system *L*_*1*_. The ESS of system *L*_*1*_ is that third-party evaluators tend to choose the *TE* strategy, and government regulators tend to choose the *NS* strategy. According to the two-dimensional dynamic system *L*_*1*_, the sum values of the equilibrium points of Jacobian matrix *J*_*1*_ are obtained. Furthermore, the local stability of system *L*_*1*_ was determined, as shown in Table 3.

**Table 3:** Local stability analysis of Case 3 and Case 4.

Balance point	Case3			_Case4_		
	${\text{tr}}J$	$\det J$	Local stability	${\text{tr}}J$	$\det J$	Local stability
$(0,0)$	$\pm$	$-$	Saddle point	$\pm$	$-$	Saddle point
$(0,1)$	$+$	$+$	Instability point	$\pm$	$-$	Saddle point
$(1,0)$	$-$	$+$	ESS	$+$	$+$	Instability point
$(1,1)$	$\pm$	$-$	Saddle point	$-$	$+$	ESS
Case 4.: When $C_{t} - C_{f} + \alpha R_{r} < F_{t} < \frac{{C_{t} - C_{f} + \alpha R_{r} }}{\beta }$ and $R_{g} > C_{g}$ at the same time, $(1,1)$ is the only stable point of system *L*_*1*_. The ESS of system *L*_*1*_ is that third-party evaluators tend to choose the *TE* strategy, and government regulators tend to choose the *AS* strategy. According to the two-dimensional dynamic system *L*_*1*_, the sum values of the equilibrium points of Jacobian matrix *J*_*1*_ are obtained. Moreover, the local stability of system *L*_*1*_ was determined, as shown in Table 3.

## Construction and analysis of the public participation model

### Model construction

In supervising pension PPP projects, government regulators often need to devote more resources to supervise third-party evaluations due to the complexity of the project organization, high concealment of rent-seeking behavior, and information asymmetry. From the stability analysis of the evolutionary strategies in Case 1 and Case 3 above, it can be seen that: (1) when government regulators’ revenue choosing the *AS* strategy is less than that of choosing the *NS* strategy ($R_{{\text{g}}} < C_{g} - \beta F_{g}$), the phenomenon of *NS* will occur; (2) when government regulators are not sufficiently penalizing third-party evaluators, the phenomenon of *FE* will occur.

To effectively solve this problem, the government should allow the public to directly or indirectly participate in the supervision of pension PPP project service quality to eradicate negative regulatory phenomena caused by insufficient regulatory resources, and then increase or decrease future cooperation opportunities, according to third-party evaluators’ public reputations. Therefore, the two factors of public participation level and third-party evaluators’ reputation are introduced into the game process of the two parties to form a new evolutionary game relationship, trying to verify their impact on the strategic choices of third-party evaluators.

For public participation, the setting of the other parameters is as follows.

$\lambda$, where $0 < \lambda < 1$ is the degree of public participation. $\lambda R_{1}$ is third-party evaluators’ additional benefit brought by the government, increasing cooperation opportunities when third-party evaluators gain the public’s trust and good reputation by choosing the *TE* strategy. $\lambda R_{2}$ is the additional loss of third-party evaluators suffered by the government, reducing their cooperation opportunities when third-party evaluators gain a bad reputation due to *FE* behavior exposure, and refers to government regulators’ accountability and penalty loss from their superiors due to being reported for *NS* by the public.

Based on the above assumptions, the evolutionary game payment matrix between third-party evaluators and government regulators with public participation is shown in Table 4.

**Table 4:** Evolutionary game payment matrix in the case of public participation.

Third-party evaluators	Government regulators	
	*AS* ($y$)	*NS* ($1 - y$)
*TE* ($x$)	$R_{t} - C_{t} + \lambda R_{1}$, $R_{g} - C_{g}$	$R_{t} - C_{t} + \lambda R_{1}$, $0$
*FE* ($1 - x$)	$R_{t} { + }\alpha R_{r} - C_{f} - \lambda R_{2} - F_{t}$,$R_{g} - C_{g}$	$R_{t} { + }\alpha R_{r} - C_{f} - \lambda R_{2} - (\lambda + \beta )F_{{\text{t}}}$,$- (\lambda + \beta )F_{g}$

### Evolution strategy stability analysis

According to the Malthusian dynamic equation^36^, the replicator dynamic equation for third-party evaluators can be obtained as

8 $$\frac{dx}{{dt}} = x(1 - x)[y(1 - \lambda - \beta )F_{t} + \lambda (R_{{1}} + R_{{2}} + F_{t} ) - (C_{t} - C_{f} + \alpha R_{{\text{r}}} - \beta F_{t} )]$$

Similarly, the replicator dynamic equation for government regulators is

9 $$\frac{dy}{{dt}} = y(1 - y)[R_{g} - C_{g} + (\lambda + \beta )F_{g} - x(\lambda + \beta )F_{g} ]$$

Therefore, in the PPP model, the evolution of third-party evaluators and government regulators’ behavioral strategies can be described by the differential equation system *L*_*2*_ consisting of the replicator dynamic Eqs. (8) and (9). By analyzing the stable point of system *L*_*2*_, five equalization points can be obtained:$$(0,0)$$,$$(0,1)$$$$(1,0)$$,$$(1,1)$$, and $$F_{5} \left( {\frac{{R_{{\text{g}}} - C_{g} + (\lambda + \beta )F_{g} }}{{(\lambda + \beta )F_{g} }},\frac{{C_{t} - C_{f} + \alpha R_{r} - \beta F_{t} - \lambda (R_{1} + R_{2} + F_{t} )}}{{(1 - \lambda - \beta )F_{t} }}} \right)$$.

According to the method proposed by Friedman^38^, the Jacobian matrix of the differential equation system composed of Eqs. (8) and (9) is

$$J_{2} = \left[ {\begin{array}{ll} {(1 - 2x)[y(1 - \lambda - \beta )F_{t} + \lambda (R_{1} + R_{2} + F_{t} ) - C_{t} + C_{f} - \alpha R_{r} + \beta F_{t} ]} & \quad {{\text{x}}(1 - x)(1 - \lambda - \beta )F_{t} } \\ { - y(1 - y)(\lambda + \beta )F_{g} } & \quad {(1 - 2y)[R_{g} - C_{g} + (1 - x)(\lambda + \beta )F_{g} ]} \\ \end{array} } \right]$$

If the following two conditions are satisfied simultaneously, the equilibrium point of the replicated dynamic equation is the ESS.

1. $${\text{tr}}J = (1 - 2x)[y(1 - \lambda - \beta )F_{t} + \lambda (R_{1} + R_{2} + F_{t} ) - C_{t} + C_{f} - \alpha R_{r} + \beta F_{t} ] + (1 - 2y)[R_{g} - C_{g} + (1 - x)(\lambda + \beta )F_{g} ] < 0$$ (Trace condition);
2. $\det = (1 - 2x)[y(1 - \lambda - \beta )F_{t} + \lambda (R_{1} + R_{2} + F_{t} ) - C_{t} + C_{f} - \alpha R_{r} + \beta F_{t} ](1 - 2y)[R_{g} - C_{g} + (1 - x)(\lambda + \beta )F_{g} ] + xy(1 - x)(1 - y)(1 - \lambda - \beta )(\lambda + \beta )F_{t} F_{g} > 0$ (Jacobian determinant condition).

Next, the ESS of system *L*_*2*_ is analyzed in four cases as follows.

Case 5.: When $F_{t} < C_{t} - C_{f} + \alpha R_{r} ,R_{g} < C_{g} - (\lambda + B)F_{g} ,\lambda < \min \left( {\frac{{C_{t} - C_{f} + \alpha R_{r} - F_{{\text{t}}} }}{{R_{{1}} + R_{{2}} }},\frac{{C_{g} - R_{g} - \beta F_{{\text{g}}} }}{{F_{g} }}} \right)$ at the same time, $(0,0)$ is the only stable point of system *L*_*2*_. The ESS of system *L*_*2*_ is that third-party evaluators tend to choose the *FE* strategy and government regulators tend to choose the *NS* strategy. According to the two-dimensional dynamic system *L*_*2*_, the sum values of the equilibrium points of the Jacobian matrix *J*_*2*_ are obtained, and the local stability of system *L*_*2*_ is determined, as shown in Table 5.

**Table 5:** Local stability analysis of Case 5 and Case 6.

Balance point	Case 5			Case 6		
	${\text{tr}}J$	$\det J$	Local stability	${\text{tr}}J$	$\det J$	Local stability
$(0,0)$	$-$	$+$	ESS	$\pm$	$-$	Saddle point
$(0,1)$	$\pm$	$-$	Saddle point	$-$	$+$	ESS
$(1,0)$	$\pm$	$-$	Instability point	$+$	$+$	Instability point
$(1,1)$	$+$	$+$	Saddle point	$\pm$	$-$	Saddle point
Case 6.: When $F_{t} < C_{t} - C_{f} + \alpha R_{r} ,R_{g} > C_{g}$, and $0 < \lambda < \, \frac{{C_{t} - C_{f} + \alpha R_{r} - F_{t} }}{{R_{{1}} + R_{{2}} }}$ at the same time, $(0,1)$ is the only stable point of system *L*_*2*_. The ESS of system* L*_*2*_ is that third-party evaluators tend to choose the *FE* strategy and government regulators tend to choose the *AS* strategy. According to the two-dimensional dynamic system *L*_*2*_, the sum values of the equilibrium points of the Jacobian matrix *J*_*2*_ are obtained, and the local stability of system *L*_*2*_ is determined, as shown in Table 5.
Case 7.: When $C_{t} - C_{f} + \alpha R_{r} < F_{t} < \frac{{C_{t} - C_{f} + \alpha R_{r} }}{\beta },R_{g} < C_{{\text{g}}} - (\lambda + \beta )F_{g}$, and $\frac{{C_{t} - C_{f} + \alpha R_{r} - \beta F_{t} }}{{R_{1} + R_{2} + F_{{\text{t}}} }} < \lambda < \frac{{C_{{\text{g}}} - R_{g} - \beta F_{g} }}{{F_{g} }}$ at the same time, $(1,0)$ is the only stable point of system *L*_*2*_. The ESS of system* L*_*2*_ is that third-party evaluators tend to choose the *TE* strategy and government regulators tend to choose the *NS* strategy. According to the two-dimensional dynamic system *L*_*2*_, the sum values of the equilibrium points of the Jacobian matrix *J*_*2*_ are obtained, and the local stability of system *L*_*2*_ is determined, as shown in Table 6.

**Table 6:** Local stability analysis of Case 7 and Case 8.

Balance point	Case 7			_Case 8_		
	${\text{tr}}J$	$\det J$	Local stability	${\text{tr}}J$	$\det J$	Local stability
$(0,0)$	$\pm$	$-$	Saddle point	$\pm$	$-$	Saddle point
$(0,1)$	$+$	$+$	Instability point	$\pm$	$-$	Saddle point
$(1,0)$	$-$	$+$	ESS	$+$	$+$	Instability point
$(1,1)$	$\pm$	$-$	Saddle point	$-$	$+$	ESS
Case 8.: When $C_{t} - C_{f} + \alpha R_{r} < F_{t} < \frac{{C_{t} - C_{f} + \alpha R_{r} }}{\beta },R_{g} > C_{g}$, and $0 < \lambda < \frac{{C_{t} - C_{f} + \alpha R_{r} - \beta F_{t} }}{{R_{1} + R_{2} + F_{{\text{t}}} }}$ at the same time,$(1,1)$ is the only stable point of system *L*_*2*_. The ESS of system* L*_*2*_ is that third-party evaluators tend to choose the *FE* strategy and government regulators tend to choose the *AS* strategy. According to the two-dimensional dynamic system *L*_*2*_, the sum values of the equilibrium points of the Jacobian matrix *J*_*2*_ are obtained, and the local stability of system *L*_*2*_ is determined, as shown in Table 6.

## Numerical analysis

Since pension PPP projects and third-party evaluations are still in their infancy in China, it is quite difficult to obtain relevant data. To better describe the evolution of third-party evaluators and government regulators’ strategic choices under the above two different conditions, we draw on the numerical examples commonly used by many scholars in applying evolutionary game theory^39,40^. To make the simulation results more scientific and objective, it is assumed that a Chinese government regulator entrusts a third-party evaluator to regularly evaluate the service quality provided by the local pension PPP projects, there are 100 beds in the PPP project center of an old-age institution which collect the elderly with good evaluation ability using market payment. To verify that public participation can effectively prevent third-party evaluators’ rent-seeking behavior, it is assumed that the probability of third-party evaluators’ rent-seeking takes a more considerable value, that is, $\alpha = 0.6$. Although the parameter assignment has absolute randomness, it does not affect the simulation results. This section verifies the eight evolutionary stability strategies and discusses the effects of $F_{t}$, $\lambda$, $R_{1}$, and $R_{2}$ on the evolution results.

### Verification of evolutionary stability strategy case

Suppose that $x = 0.1,\;y = 0.1$,$x = 0.2,\;y = 0.2$,$x = 0.5,\;y = 0.5$,$x = 0.6,\;y = 0.6$, and $x = 0.9,\;y = 0.9$ are five different initial ratios randomly assigned to each game player in the game. The simulation is shown in figures that the horizontal axis represents *the possibility of TE* or *AS* on both sides of the game, while the vertical axis represents the time in months.

**Figure 1:**
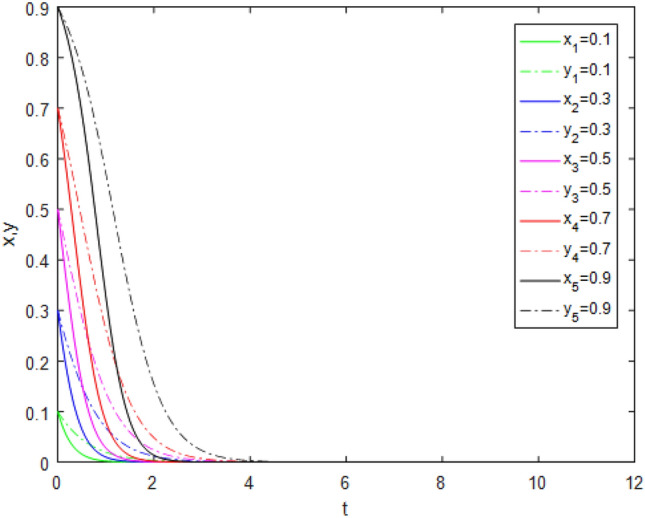
Simulation results for Case 1.

**Figure 2:**
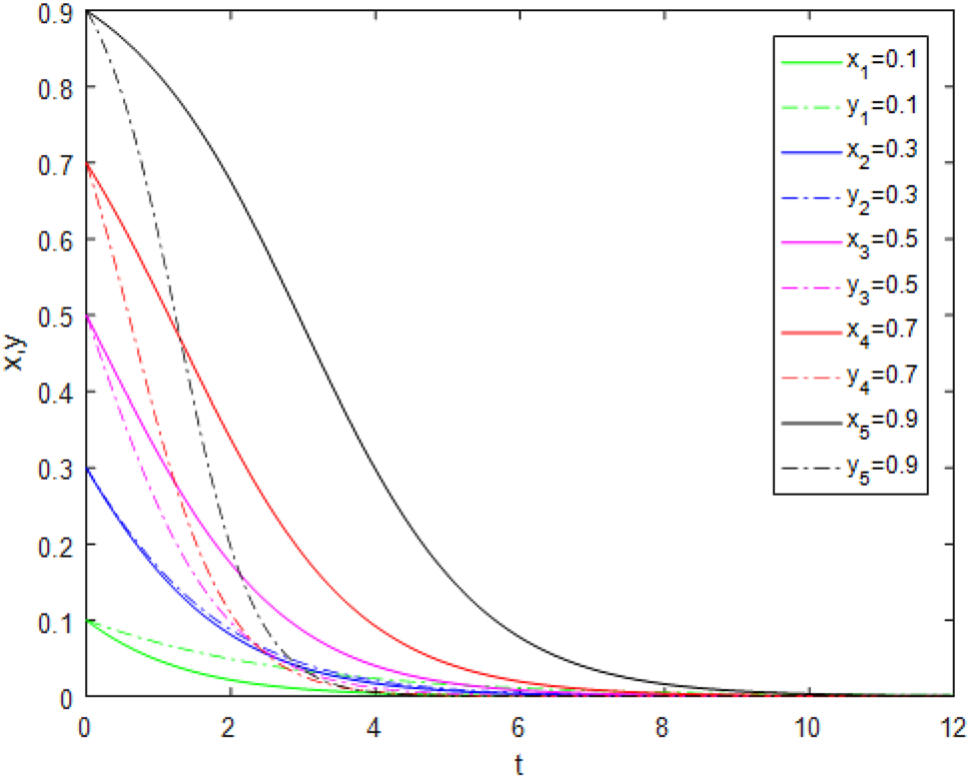
Simulation results for Case 5.

Suppose that simulated values of parameters in the Case 1 and Case 5 of evolutionary stabilization strategies are listed in Table 7. The simulation is shown in Figs. 1, 2.

**Table 7:** Simulated values of parameters in the Case 1and Case 5 of evolutionary stabilization strategies.

Case	$R_{g}$	$C_{g}$	$F_{g}$	$\beta$	$\lambda$	$\alpha$	$R_{r}$	$F_{t}$	$C_{t}$	$C_{f}$	$R_{1}$	$R_{2}$	Diagram
Case1	2	4	2	0.2	**0**	0.6	2	1	3	1	**0**	**0**	Figure 1
Case 5	2	4	2	0.2	**0.7**	0.6	2	1	3	1	**1**	**2**	Figure 2

Significant values are in bold.

Both Figs. 1 and 2 show that the game system always tends to point under different initial ratios, consistent with the analysis of Case 1 and Case 5 respectively. In Case 1 and Case 5, the government’s punishment for third-party evaluator violations is minimal. Comparing Figs. 1 and 2, it can be seen that Fig. 2 shows the evolution time which *x* and *y* are close to 0, is longer when the values of $\lambda$, $R_{1}$ and $R_{2}$ increase. Although public participation in Case 5 is very high which is difficult to achieve in reality, it does not constitute a deterrent effect for third-party evaluators. Therefore, third-party evaluators choose the *FE* strategy. This shows that even if public participation is high, public participation mechanisms will be ineffective if there is no suitable punishment mechanism. Since the cost of government supervision is far greater than the benefits of supervision, and the penalties imposed by superiors on government regulators for failure to supervise are minimal, government regulators choose the *NS* strategy. At this time, the interaction between the two sides develops to the worst balance point, the government supervision mechanism is in the "invalid" state, and public participation cannot promote the reliability of third-party evaluation institutions.

**Figure 3:**
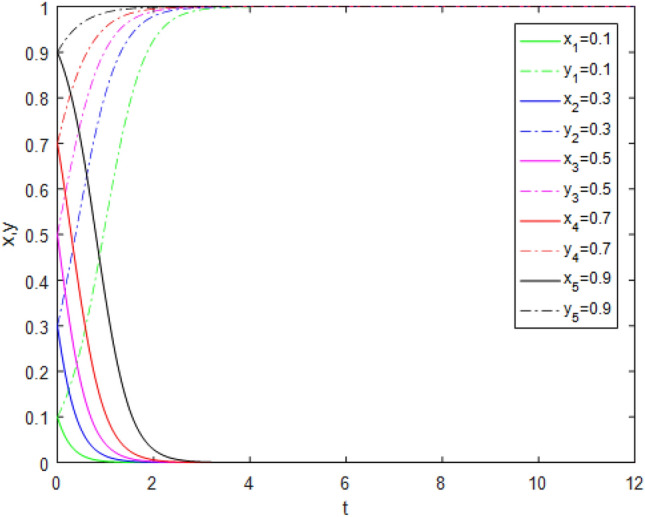
Simulation results for Case 2.

**Figure 4:**
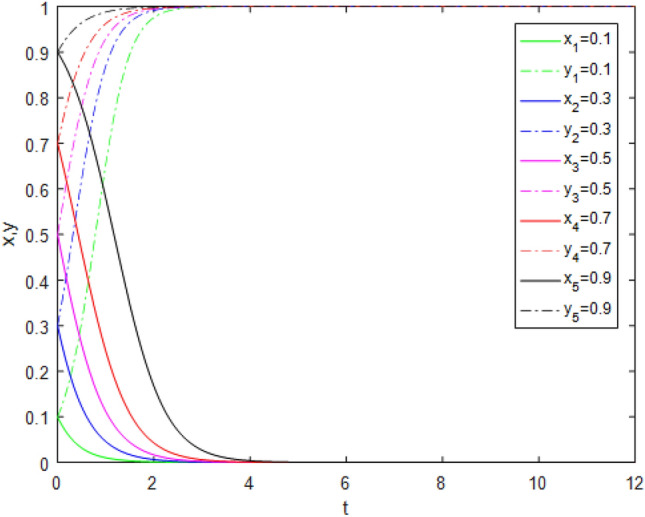
Simulation results for Case 6.

Suppose that simulated values of parameters in the Case 2 and Case 6 of evolutionary stabilization strategies are listed in Table 8. The simulation is shown in Figs. 3, 4.

**Table 8:** Simulated values of parameters in the Case 2 and Case 6 of evolutionary stabilization strategies.

Case	$R_{g}$	$C_{g}$	$F_{g}$	$\beta$	$\lambda$	$\alpha$	$R_{r}$	$F_{t}$	$C_{t}$	$C_{f}$	$R_{1}$	$R_{2}$	Diagram
Case 2	4	2	2	0.2	**0**	0.9	2	1	3	1	**0**	**0**	Figure 3
Case 6	4	2	2	0.2	**0.7**	0.9	2	1	3	1	**1**	**2**	Figure 4

Significant values are in bold.

Both Figs. 3 and 4 show that the game system always tends to point $(0,1)$ under different initial ratios, consistent with the analysis of Case 2 and Case 6 respectively. In Cases 2 and Case 6,the government’s punishment for third-party evaluators’violations is small, as in Cases 1 and 5. From the comparison of Figs. 3 and 4, it can be found that, when the values of $\lambda$, $R_{1}$ and $R_{2}$ increase, the evolution time which *x* is close to 0, is longer. Although public participation in Case 6 is as high as in Case 5, it also does not constitute a deterrent effect for third-party evaluators. Therefore, third-party evaluators choose the *FE* strategy. In comparison, the government regulatory revenue is far greater than the regulatory cost, which arouses government regulators’ enthusiasm, so government regulators choose the *AS* strategy. At this time, the interaction between the two sides develops into a bad "locked"state. Although government regulators actively supervise and public participation is also high, the punishment mechanism is not perfect, and third-party evaluators still fail.

**Figure 5:**
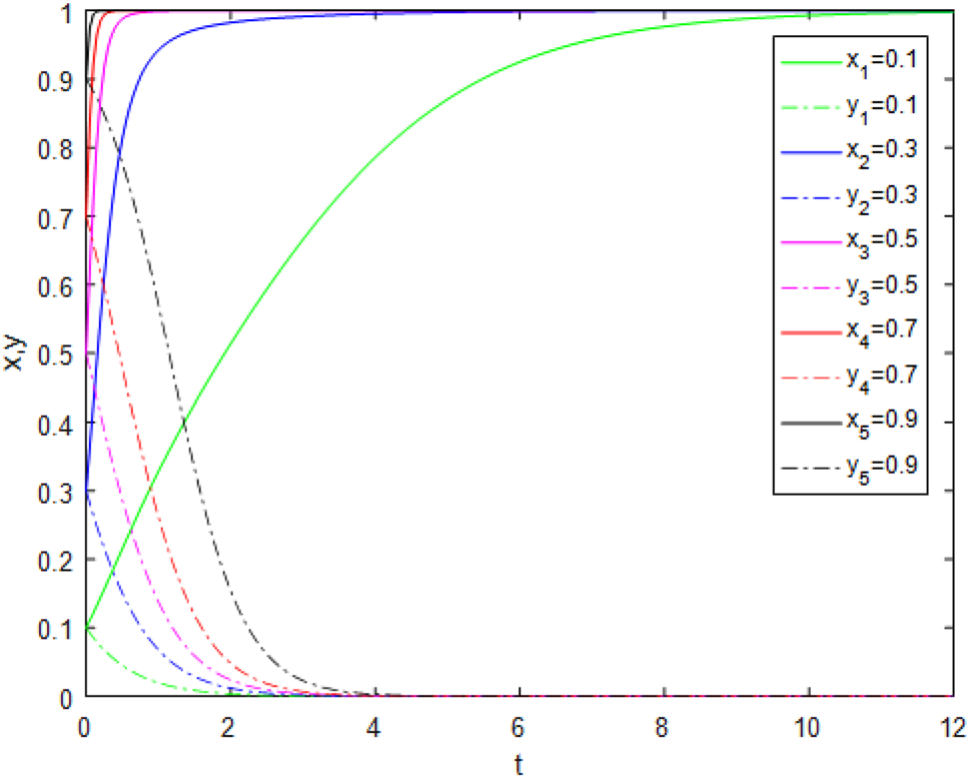
Simulation results for Case 3.

**Figure 6:**
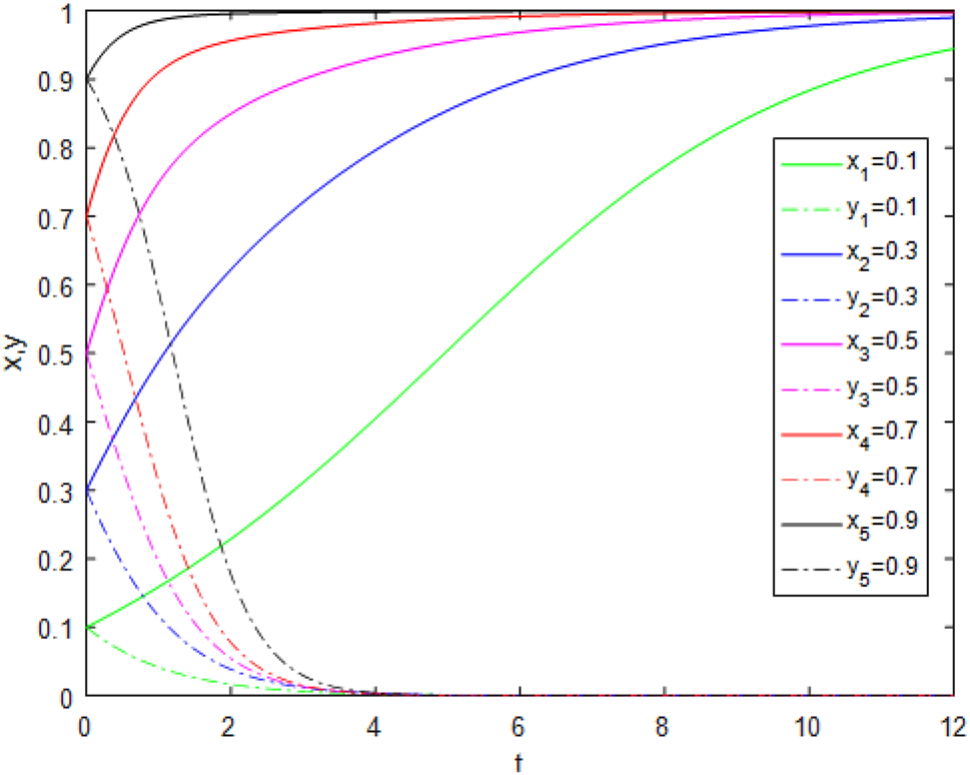
Simulation results for Case 7.

Suppose that simulated values of parameters in the Case 3 and Case 7 of evolutionary stabilization strategies are listed in Table 9. The simulation is shown in Figs. 5, 6.

**Table 9:** Simulated values of parameters in Case 3 and Case 7 of evolutionary stabilization strategies.

Case	$R_{g}$	$C_{g}$	$F_{g}$	$\beta$	$\lambda$	$\alpha$	$R_{r}$	$F_{t}$	$C_{t}$	$C_{f}$	$R_{1}$	$R_{2}$	Diagram
Case 3	2	4	2	0.2	**0**	0.9	2	**22**	3	1	**0**	**0**	Figure 5
Case 7	2	4	2	0.2	**0.4**	0.9	2	**5**	3	1	**1**	**2**	Figure 6

Significant values are in bold.

Both Figs. 5 and 6 show that the game system always tends to point $(1,0)$ under different initial ratios, consistent with the analysis of Case 3 and Case 7 respectively. In Case 3, the government strongly punishes third-party evaluators’ illegal behavior. Third-party evaluators consciously choose the *TE* strategy, even if government regulators do not supervise. However, it is easy to cause excessive punishment and affect third-party evaluators’ enthusiasm to undertake assessment business. In Case 7, the government moderately punishes the third-party evaluators’ illegal behavior. Under public participation and reputation incentives, third-party evaluators will actively choose the *TE* strategy, even if government regulators do not supervise. Therefore, in the case of limited government regulatory resources, public participation combined with appropriate punishment mechanisms can reduce government regulators’ burden and improve third-party evaluator evaluation reliability.

**Figure7:**
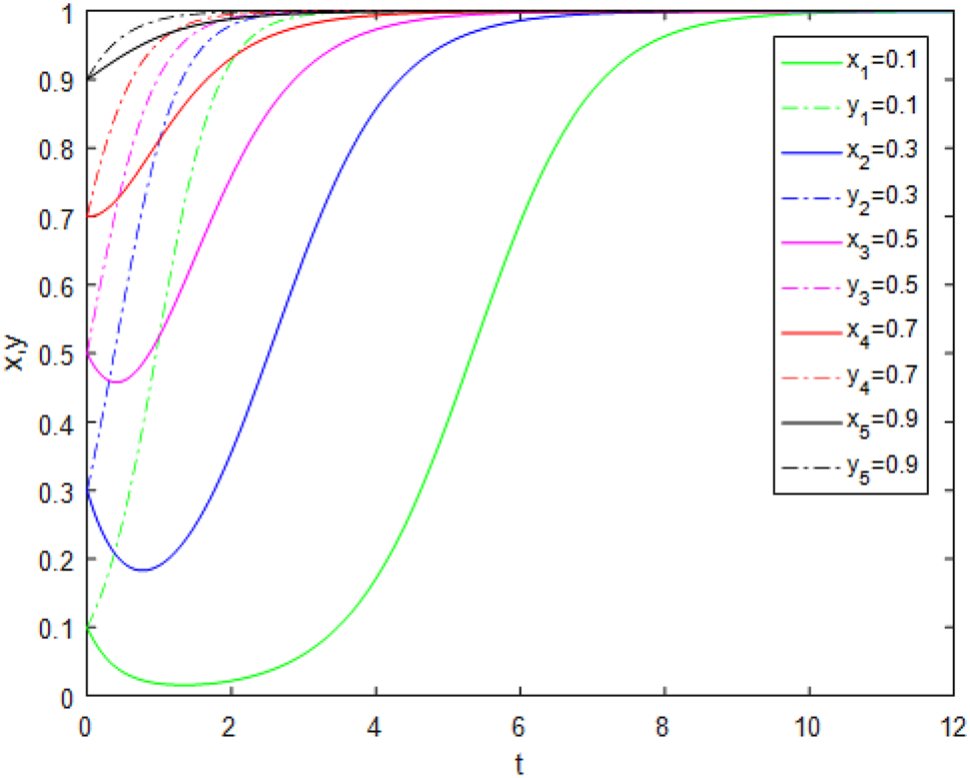
Simulation results for Case 4.

**Figure 8:**
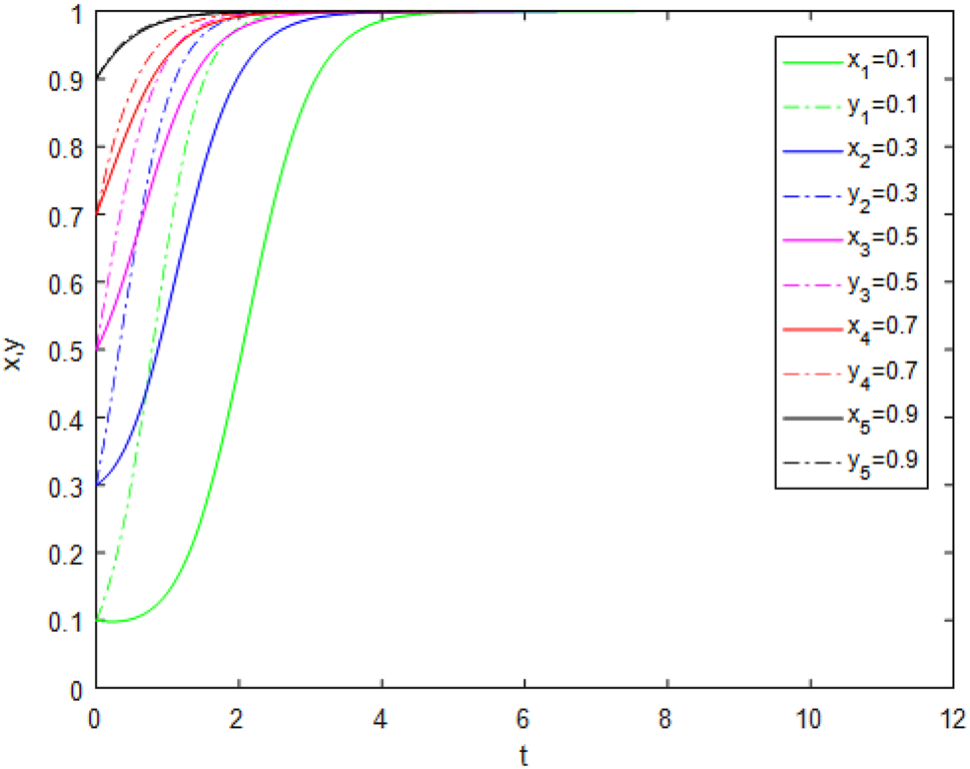
Simulation results for Case 8.

Suppose that simulated values of parameters in Case 4 and Case 8 of evolutionary stabilization strategies are listed in Table 10. The simulation is shown in Figs. 7, 8.

**Table 10:** Simulated values of parameters in Case 4 and Case 8 of evolutionary stabilization strategies.

Case	$R_{g}$	$C_{g}$	$F_{g}$	$\beta$	$\lambda$	$\alpha$	$R_{r}$	$F_{t}$	$C_{t}$	$C_{f}$	$R_{1}$	$R_{2}$	Diagram
Case 4	4	2	2	0.2	**0**	0.9	2	5	3	1	**0**	**0**	Figure 7
Case 8	4	2	2	0.2	**0.3**	0.9	2	5	3	1	**1**	**2**	Figure 8

Significant values are in bold.

Both Figs. 7 and 8 show that the game system always tends to point (1,1) under different initial ratios, consistent with the analysis of Cases 4 and 8 respectively. Comparing Figs. 7 and 8, although both show that the "ideal" state point (1,1) of the game is reached, the game system achieves the ideal goal faster under Case 8 from a time perspective. This shows that public participation can promote a faster and more benign system transformation. At this time, regulatory resources are fully utilized, the quality of pension services is effectively controlled, social benefits are maximized, and sustainable and high-quality development of pension PPP projects is realized.

### Contrastive analysis of evolution trajectory

**Figure 9:**
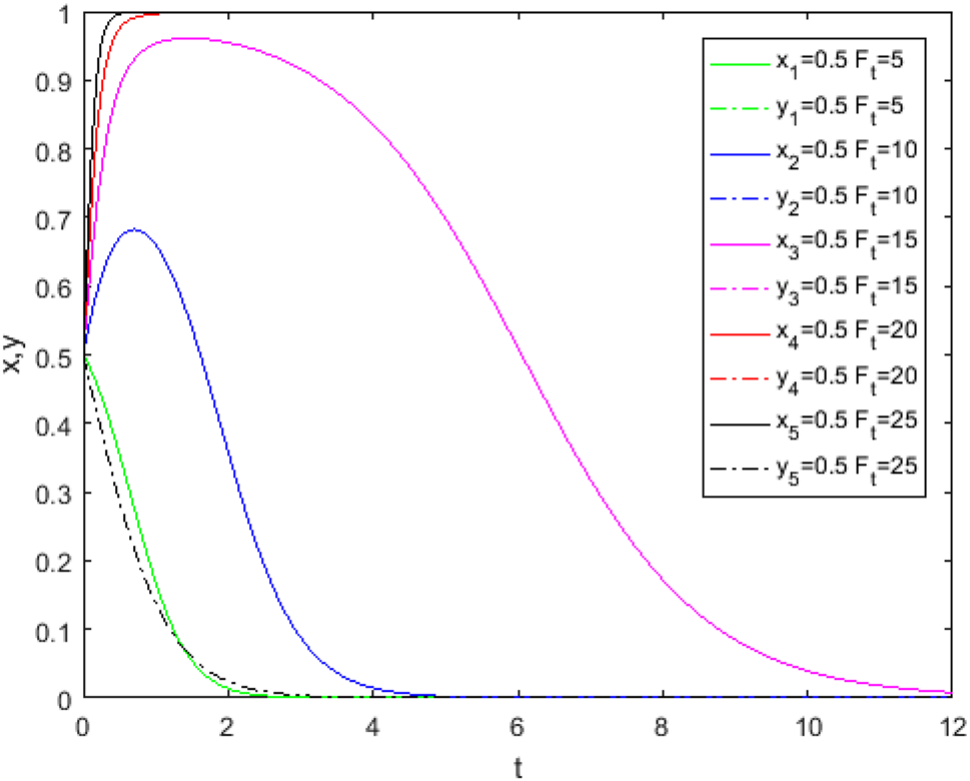
Evolution track of the change of $F_{t}$ under Case 1.

**Figure 10:**
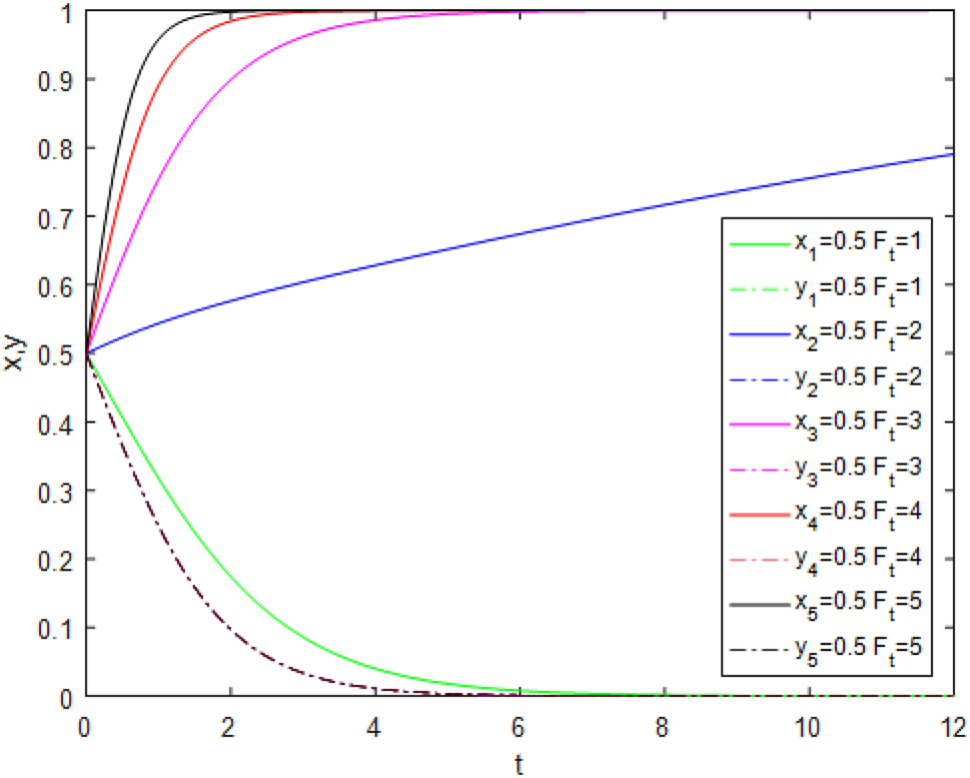
Evolution track of the change of $F_{t}$ under Case 5.

**Figure 11:**
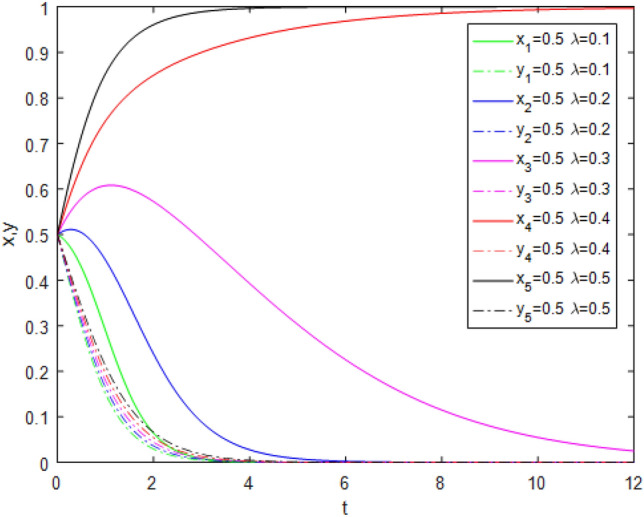
Evolution track of the change of $\lambda$ under Case 7.

**Figure 12:**
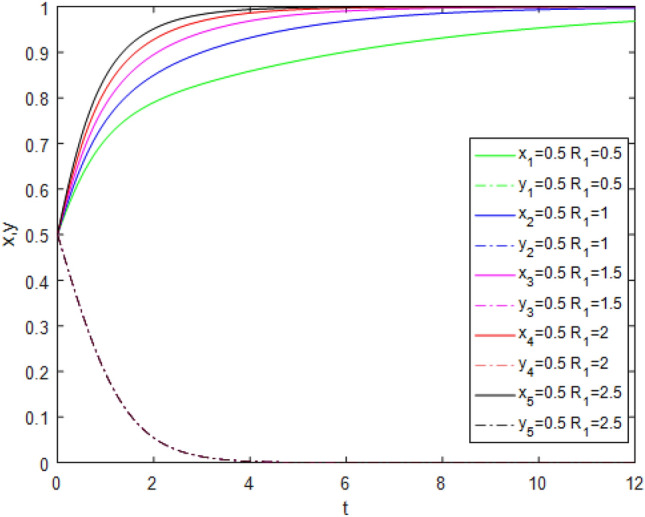
Evolution track of the change of $R_{1}$ under Case 7.

Suppose that the initial condition is $x = 0.5,\;{\text{y}} = 0.5$. First, based on the parameter values under Case1 (i.e., the parameter assignment in Fig. 1), $F_{{\text{t}}}$ is assumed as a variable, and the MATLAB simulation program is then carried out. The results are shown in Fig. 9. Second, based on the parameter values in Case 5 (i.e., the parameter assignment in Fig. 2), $F_{{\text{t}}}$ is assumed as a variable, and the MATLAB simulation program is performed. The results are shown in Fig. 10. Finally, based on the parameter values under Case 7, that is, the parameter assignment in Fig. 6, $\lambda$ and $R_{1}$ are assumed as variables, and the simulation is compiled. The results are shown in Figs. 11, 12.

Figure 9 shows the impact of the penalty parameter $F_{t}$ changing from 5 to 25 on the system evolution under Case1 conditions. The game system still tends to be stable at point (0,0) when the penalty parameter $F_{t}$ changes from 5 to 15, while it evolves from point (0,0) to point (1,0) only when $F_{{\text{t}}} > 15$. It takes such heavy penalties to effectively restrict third-party evaluators for violations without public participation.

Figure 10 shows the impact of penalty parameters $F_{t}$ changing from 1 to 5 in the system evolution under Case 5 conditions. The game system still tends to be stable at point (0, 0) when $F_{t} < 2$, while it evolves from point (0,0) to point (1,0) only when $F_{{\text{t}}} \ge 2$. That is, in the case of high levels of public participation, as long as the relatively light punishment can effectively limit violations of third-party evaluators.

It can also be seen from Figs. 9 and 10 that with the increase in $F_{t}$**,** the acceleration of the system converging to point (1,0) gradually decreases. This may be because excessive punishment, to a certain extent, inhibits the enthusiasm of third-party evaluators to adopt the *TE* strategy, which leads to a reduction in the marginal effect of punishment measures. Therefore, the punishment mechanism should be reasonable to ensure that its "positive incentive" effect is brought into full play.

Figures 9 and 10 indicate that when government regulators choose the *NS* strategy, public participation can avoid government regulators’ insufficient punishment and promote third-party evaluators to choose the *FE* strategy to a certain extent. This indirectly proves that public participation promotes the reliability of third-party evaluator evaluations.

Figure 11 shows the impact of the public participation coefficient $\lambda$ changing from 0.1 to 0.5 on system evolution under Case 7 conditions. The game system still tends to be stable at point (0, 0) when the public participation coefficient $\lambda$ changes from 0.1 to 0.3, while it starts to evolve positively from point (0,0) to point (1,0) only when $\lambda > 0.3$. Figure 11 also shows that the higher the level of public participation, the slower the government regulators tend to choose the *AS* strategy. This indicates that in the case of public participation, with the improvement of public participation, even if government regulators fail to supervise, third-party evaluators will choose the *TE* strategy. This directly proves that public participation promotes the reliability of third-party evaluations. However, promoting public participation is not easy. It requires a series of government supporting mechanisms.

Figure 12 shows the impact of reputation incentive parameters $R_{1}$ changing from 0.5 to 2.5 on system evolution under Case 7 conditions. With the strengthening of the reputation incentive, the game system tends to point (1,0) progressively faster. This indicates that when government regulators choose the *NS* strategy, third-party evaluators tend to choose the *TE* strategy because of the positive strengthening of reputation incentives with public participation. This directly proves that public participation promotes the reliability of third-party evaluator evaluations. However, reputational incentives cannot be strengthened excessively.A reasonable reputational mechanism should be established to ensure the maximization of the marginal effects of reputational incentives.

Considering the identicality of the parameter trends in other cases, they are not compared in this study.

### Simulation case

**Figure 13:**
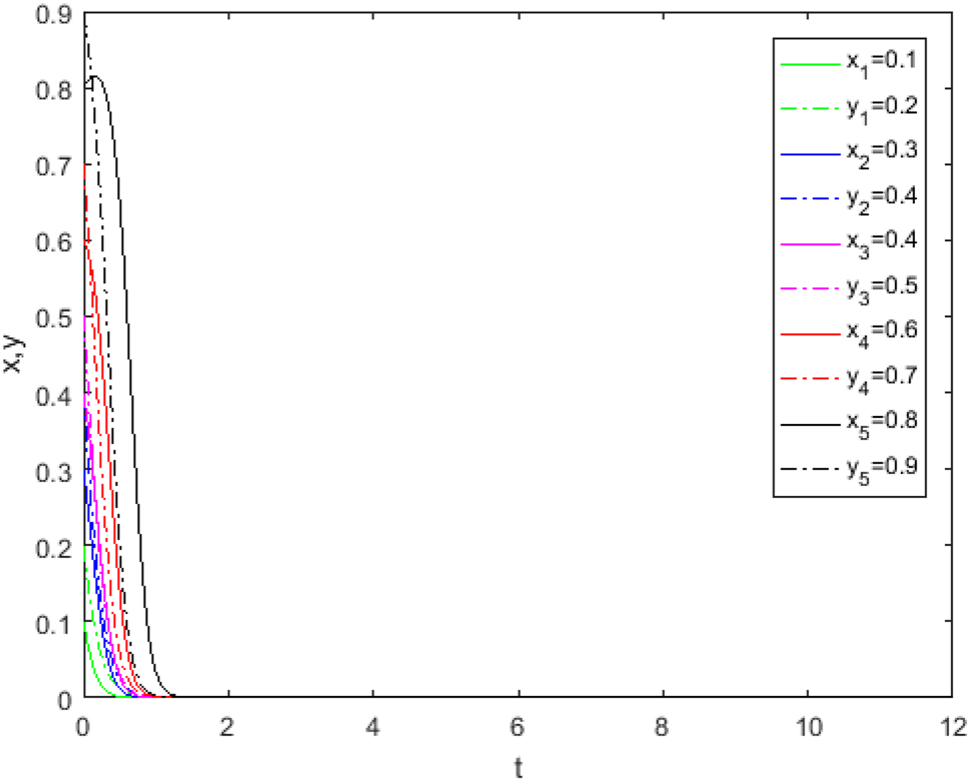
Evolution track without public participation.

**Figure 14:**
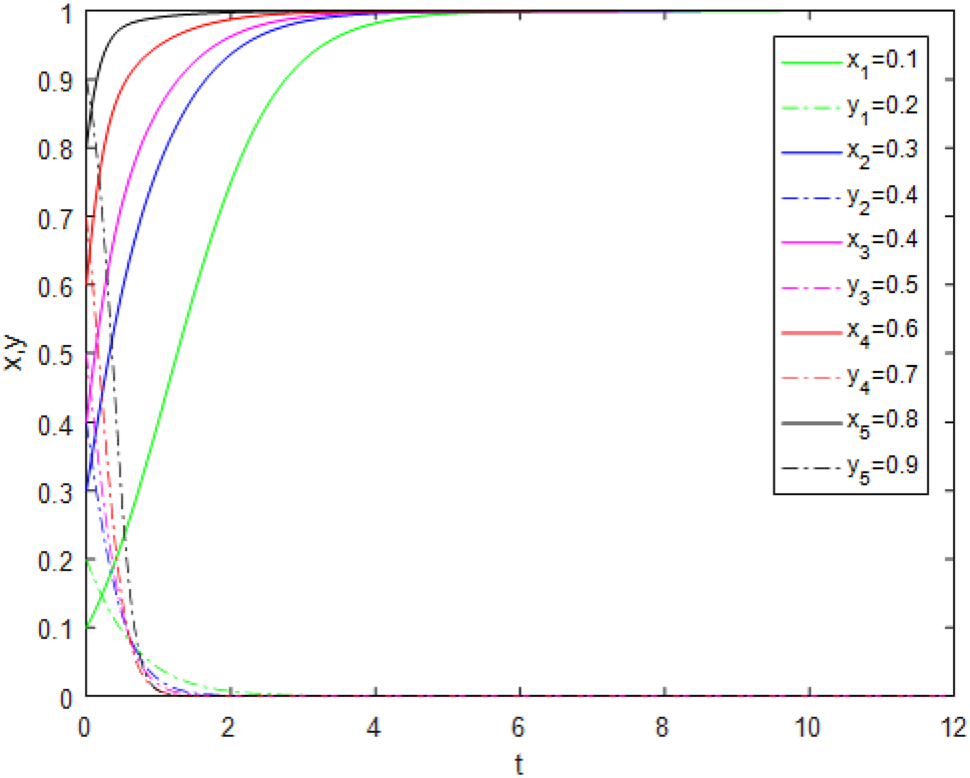
Evolution track with public participation.

Through the survey, it is assumed that a third-party evaluator in a province of China obtains the service quality evaluation authority of 20 pension PPP projects in the province through bidding, and the average income of each project *R*_*t*_ is 20,000 yuan. The value assignment of other parameters without public participation and with public participation is shown in Tables 11 and 12. To better verify the correctness of the game model, suppose that $x = 0.1,\;y = 0.2$,$x = 0.3,\;y = 0.4$,$x = 0.4,\;y = 0.5$, $x = 0.6,\;y = 0.7$, and $x = 0.8,\;y = 0.9$ are five different initial ratios randomly assigned to each game player in the game. The simulation is shown in Figs. 13, 14.

**Table 11:** Simulated values of parameters without public participation (unit: RMB 1000).

Parameters	$R_{g}$	$C_{g}$	$F_{g}$	$\beta$	$\lambda$	$\alpha$	$R_{r}$	$F_{t}$	$C_{t}$	$C_{f}$	$R_{1}$	$R_{2}$	Diagram
Values	12	20	10	0.3	**0**	0.4	5	14	15	5	**0**	**0**	Figure 13

Significant values are in bold.

**Table 12:** Simulated values of parameters with public participation (unit: RMB 1000).

Parameters	$R_{g}$	$C_{g}$	$F_{g}$	$\beta$	$\lambda$	$\alpha$	$R_{r}$	$F_{t}$	$C_{t}$	$C_{f}$	$R_{1}$	$R_{2}$	Diagram
Values	12	20	10	0.3	**0.3**	0.4	5	14	15	5	**4**	**5**	Figure 14

Significant values are in bold.

When $R_{g} < C_{g}$, Fig. 13 shows that the game system tends to be stable at point (0, 0) without public participation while Fig. 14 shows that the game system tends to be stable at point (0, 1) with public participation. It shows that in the case of public participation, even if the government chooses the *NS* strategy, the third-party evaluation agency will also choose the *TE* strategy under the influence of reputation gains. This again proves that public participation and reputation incentive have a greater impact on the behavior strategy choice of third-party evaluator.

## Conclusions and implications

### Conclusions

Based on the information asymmetry and the players’ bounded rationality, this study uses evolutionary game theory to establish a game model between government regulators and third-party evaluators under two different conditions, while comparing and analyzing the evolutionary trends of third-party evaluators’ behavior strategies. Combined with MATLAB simulation analysis, we conclude that third-party evaluators may choose the *FE* strategy without public participation because of the inducement of rent-seeking or insufficient government’s punishment when the regulatory revenue of the government regulatory agencies is less than the regulatory cost. In contrast, in the case of public participation, the *TE* strategy is chosen with an improvement in the level of public participation or an increase in reputation incentive. When the cost of government supervision cannot be reduced and government supervision resources are limited, it is necessary to improve public participation by guiding it to reduce the probability of third-party evaluators adopting the *FE* strategy based on rent-seeking income. Simultaneously, it is also necessary to establish a reputation mechanism that spurs third-party evaluators to improve credibility, scientific, and evaluation accuracy. This suggests the construction and improvement of a third-party evaluation system, which shows that the construction of the service quality supervision system in China’s pension PPP project has a large operating space.

### Implications

To promote the reliability of third-party evaluation with public participation and improve pension PPP project sustainability, there are several managerial implications for decision-makers.

The first is to establish and improve laws and regulations encouraging public participation. The Chinese government should guide the public to actively participate in pension PPP project supervision by improving the public participation system and encouraging the public to coordinate public interest and social governance. Simultaneously, the government should actively establish public participation organizations, encourage and recognize non-profit public participation organizations, and guide the public through grassroots self-governing organizations, unit trade unions, social welfare organizations, and other types of collective auction participation. Internet public reporting platforms, such as Weibo or WeChat Public Account, should be set up to provide convenient ways for the public to actively participate in the supervision or reporting of violations by third-party evaluators, reduce the cost and risk of public reporting, and improve regulatory efficiency.

The second is to build a pension PPP project management information system to institute information resource sharing, real-time display pension service type, charge pension PPP projects based on a public evaluation function, and minimize the information asymmetry between the supervisors and the supervised, which can provide useful detailed information to third-party evaluators, again reducing costs, and encouraging objective evaluations.

The third is to establish and improve the third-party evaluation system for pension PPP projects’ service quality. As the related service quality evaluation is gradually entrusted to third-party evaluators, the Chinese government should improve the evaluation mechanism and implementation methods for third-party evaluators, clarify the responsibilities of government regulators, and establish a third-party supervision mechanism. This should reasonably monitor the third-party evaluation system and formulate a third-party recognition method and system. By horizontally comparing third-party evaluators’ strengths, such as professional capabilities, staffing, and data processing and monitoring technologies, entry barriers are increased to ensure that only qualified third-party evaluators can obtain evaluation business.

The fourth is to establish an information disclosure mechanism and strengthen the reputation incentive mechanism. Given the professionalism and complexity of service quality evaluations of pension PPP projects, third-party evaluators have information advantages due to information asymmetry. It is necessary to make full use of big data technology and network information platforms to promptly publish third-party evaluation reports. This facilitates regulatory supervision, competition, and public involvement, and discloses when third-party evaluators violate laws or regulations. Simultaneously, reputation factors can be used to restrict third-party evaluators’ behavior and decision-making, and give full play to the public’s role in supervising third-party evaluators’ behavior.

The fifth is to improve third-party evaluators’ reward and punishment mechanisms. This model research also shows that third-party evaluators will choose the *TE* strategy when the punishment is strong enough, even if government regulators choose the *NS* strategy. Therefore, it is necessary to increase the consequences for third-party evaluators who violate regulations by suspending their qualifications and even canceling the cooperation, while also implementing joint and several liability systems for injuries to weaken their rent-seeking motivation. Of course, it is also necessary to establish an incentive mechanism to ensure the sustainability of third-party evaluators choosing the *TE* strategy to provide specific policy subsidies or increase their social recognition and trust.

## Data Availability

The data used to support the findings of this study are included within the article.
